# Supplementary material for: A cross-sectional study into the prevalence and conformational risk factors of BOAS across fourteen brachycephalic dog breeds
Source: PLoS One. 2026 Feb 18;21(2):e0340604. doi: 10.1371/journal.pone.0340604 (PMC12915975; doi:10.1371/journal.pone.0340604)
Supplement: S1 Table — (DOCX) [file pone.0340604.s002.docx]

| **Breed** | **% KC reg** | **% Male** | **% Neutered** | **Median age (year; range)** |
| --- | --- | --- | --- | --- |
| **Affenpinscher** | 98% | 39% | 13% | 3.0 (1.0 - 14.0) |
| **Boston Terrier** | 89% | 37% | 35% | 3.0 (1.0 - 10.0) |
| **Boxer** | 97% | 38% | 36% | 3.9 (1.0 - 9.8) |
| **Cavalier King Charles Spaniel** | 68% | 38% | 66% | 6.2 (1.1 - 14.0) |
| **Chihuahua** | 60% | 48% | 52% | 6.4 (1.4 - 15.0) |
| **Dogue de Bordeaux** | 91% | 43% | 16% | 2.5 (1.3 - 12.3) |
| **Griffon Bruxellois** | 91% | 52% | 36% | 6.0 (1.0 - 14.1) |
| **Japanese Chin** | 91% | 56% | 21% | 3.0 (1.1 - 13.0) |
| **King Charles Spaniel** | 99% | 49% | 15% | 4.5 (1.0 - 12.0) |
| **Maltese** | 86% | 31% | 47% | 6.0 (1.3 - 12.5) |
| **Pekingese** | 92% | 54% | 14% | 3.5 (1.1 - 15.0) |
| **Pomeranian** | 94% | 35% | 17% | 4.5 (1.0 - 13.7) |
| **Shih Tzu** | 51% | 42% | 60% | 8.0 (1.0 - 12.2) |
| **Staffordshire Bull Terrier** | 100% | 34% | 21% | 2.8 (1.0 - 15.8) |
