# Supplementary material for: A cross-sectional study into the prevalence and conformational risk factors of BOAS across fourteen brachycephalic dog breeds
Source: PLoS One. 2026 Feb 18;21(2):e0340604. doi: 10.1371/journal.pone.0340604 (PMC12915975; doi:10.1371/journal.pone.0340604)
Supplement: S2 Table — (DOCX) [file pone.0340604.s003.docx]

| Breed | Heart murmur | Patella luxation | Facial fold | Abnormal scleral show | Ectropion |
| --- | --- | --- | --- | --- | --- |
| Affenpinscher | 2.9% | 11.8% | 0% | 5.1% | 0% |
| Boston Terrier | 4.5% | 14.5% | 32.3% | 63.7% | 0% |
| Boxer | 7.2% | 0% | 57.9% | 7.4% | 50.5% |
| Cavalier King Charles Spaniel | 33.3% | 8.0% | 1.1% | 36.0% | 0% |
| Chihuahua | 7.8% | 38.0% | 0% | 34.6% | 0% |
| Dogue de Bordeaux | 3.8% | 0% | 81.4% | 0% | 52.5% |
| Griffon Bruxellois | 4.9% | 9.1% | 11.4% | 35.7% | 1.9% |
| Japanese Chin | 16.0% | 36.2% | 75.9% | 75.9% | 0% |
| King Charles Spaniel | 9.5% | 34.1% | 77.9% | 54.7% | 2.5% |
| Maltese | 6.1% | 27.3% | 0% | 2.9% | 0% |
| Pekingese | 0% | 25.5% | 79.6% | 77.8% | 0% |
| Pomeranian | 0% | 34.0% | 0% | 3.6% | 0% |
| Shih Tzu | 2.0% | 20.0% | 9.1% | 30.9% | 0% |
| Staffordshire Bull Terrier | 0% | 4.7% | 0.7% | 0.7% | 0% |
