# Supplementary material for: A cross-sectional study into the prevalence and conformational risk factors of BOAS across fourteen brachycephalic dog breeds
Source: PLoS One. 2026 Feb 18;21(2):e0340604. doi: 10.1371/journal.pone.0340604 (PMC12915975; doi:10.1371/journal.pone.0340604)
Supplement: S3 Table — (DOCX) [file pone.0340604.s004.docx]

| Breed | n | % Grade 0 | % Grade 1 | % Grade 2 | % Grade 3 |
| --- | --- | --- | --- | --- | --- |
| Pekingese | 45 | 10.9 | 34.8 | 54.3 | 0.0 |
| Japanese Chin | 46 | 17.4 | 34.8 | 45.7 | 2.2 |
| Griffon Bruxellois | 52 | 26.9 | 40.4 | 30.8 | 1.9 |
| Boston Terrier | 107 | 38.3 | 34.6 | 26.2 | 0.9 |
| Dogue de Bordeaux | 51 | 41.2 | 39.2 | 19.6 | 0.0 |
| King Charles Spaniel | 83 | 39.8 | 42.2 | 18.1 | 0.0 |
| Shih Tzu | 42 | 47.6 | 33.3 | 19.0 | 0.0 |
| Staffordshire Bull Terrier | 120 | 62.5 | 30.0 | 5.8 | 1.7 |
| Cavalier King Charles Spaniel | 73 | 68.5 | 24.7 | 6.8 | 0.0 |
| Chihuahua | 47 | 74.5 | 10.6 | 14.9 | 0.0 |
| Boxer | 79 | 78.5 | 19 | 2.5 | 0.0 |
| Affenpinscher | 69 | 78.3 | 15.9 | 5.8 | 0.0 |
| Pomeranian | 51 | 84.3 | 15.7 | 0.0 | 0.0 |
| Maltese | 32 | 96.9 | 3.1 | 0.0 | 0.0 |
