# Supplementary material for: A cross-sectional study into the prevalence and conformational risk factors of BOAS across fourteen brachycephalic dog breeds
Source: PLoS One. 2026 Feb 18;21(2):e0340604. doi: 10.1371/journal.pone.0340604 (PMC12915975; doi:10.1371/journal.pone.0340604)
Supplement: S4 Table — (A) Raw p-values from continuous data univariate analysis from one-tailed t-test and categorical variable Fisher’s exact test p-value results. Significance level is set at 0.05. (B) P-values following adjustment using Holm-Sidak method whereby alpha = 0.05 and p-value significance threshold is set at < 0.004. (DOCX) [file pone.0340604.s005.docx]

|  | 1. **Univariate Analysis: Raw P Values** | | | | | | | | |
| --- | --- | --- | --- | --- | --- | --- | --- | --- | --- |
|  | **BH** | **BL** | **BL:BH** | **TL** | **NGR** | **EWR** | **CFR** | **Nostril stenosis** | **BCS** |
| **Affenpinscher** | 0.17 | 0.22 | 0.05 | 0.12 | 0.10 | 0.09 | 0.48 | 0.07 | 0.07 |
| **Boston Terrier** | 0.38 | 0.37 | 0.27 | 0.05 | 0.04 | 0.15 | < 0.0001 | 0.0002 | 0.17 |
| **Boxer** | 0.47 | 0.38 | 0.39 | 0.33 | 0.42 | 0.03 | 0.007 | 0.02 | 0.47 |
| **CKCS** | 0.02 | 0.21 | 0.10 | 0.16 | 0.31 | 0.32 | 0.12 | 0.04 | 0.006 |
| **Chihuahua** | 0.06 | 0.45 | 0.047 | 0.01 | 0.40 | 0.35 | 0.0036 | 0.81 | > 0.999 |
| **DDB** | 0.15 | 0.10 | 0.45 | 0.38 | 0.07 | 0.45 | 0.39 | 0.35 | 0.52 |
| **Griffon** | 0.13 | 0.11 | 0.29 | 0.14 | 0.46 | 0.05 | 0.18 | 0.01 | 0.11 |
| **Japanese Chin** | 0.22 | 0.28 | 0.39 | 0.10 | 0.14 | 0.37 | 0.24 | 0.45 | 0.28 |
| **KCS** | 0.09 | 0.08 | 0.02 | 0.37 | 0.18 | 0.21 | 0.35 | 0.02 | 0.02 |
| **Pekingese** | 0.20 | 0.33 | 0.31 | 0.40 | 0.44 | 0.07 | 0.29 | 0.008 | 0.22 |
| **Pomeranian** | 0.11 | 0.38 | 0.08 | 0.26 | 0.46 | 0.37 | 0.0001 | 0.24 | 0.43 |
| **Shih Tzu** | 0.01 | 0.34 | 0.25 | 0.003 | 0.34 | 0.04 | 0.07 | 0.64 | 0.22 |
| **SBT** | 0.32 | 0.41 | 0.42 | 0.0003 | 0.007 | 0.20 | 0.06 | 0.80 | 0.03 |
|  |  |  |  |  |  |  |  |  |  |
|  | 1. **Univariate Analysis: Adjusted P Values** | | | | | | | | |
|  | **BH** | **BL** | **BL:BH** | **TL** | **NGR** | **EWR** | **CFR** | **Nostril stenosis** | **BCS** |
| **Affenpinscher** | 0.73 | 0.91 | 0.44 | 0.64 | 0.85 | 0.74 | 0.75 | 0.67 | 0.52 |
| **Boston Terrier** | 0.74 | 0.94 | 0.90 | 0.40 | 0.39 | 0.74 | 0.001 | 0.03 | 0.81 |
| **Boxer** | 0.74 | 0.94 | 0.90 | 0.80 | 0.93 | 0.33 | 0.10 | 0.20 | 0.89 |
| **CKCS** | 0.22 | 0.91 | 0.61 | 0.65 | 0.93 | 0.85 | 0.59 | 0.31 | 0.23 |
| **Chihuahua** | 0.49 | 0.94 | 0.44 | 0.10 | 0.93 | 0.85 | 0.047 | 0.99 | 1.00 |
| **DDB** | 0.73 | 0.72 | 0.90 | 0.80 | 0.55 | 0.85 | 0.75 | 0.89 | 0.89 |
| **Griffon** | 0.74 | 0.89 | 0.90 | 0.65 | 0.93 | 0.61 | 0.70 | 0.67 | 0.65 |
| **Japanese Chin** | 0.74 | 0.93 | 0.90 | 0.61 | 0.78 | 0.85 | 0.75 | 0.93 | 0.85 |
| **KCS** | 0.61 | 0.66 | 0.23 | 0.80 | 0.83 | 0.79 | 0.75 | 0.20 | 0.23 |
| **Pekingese** | 0.65 | 0.94 | 0.57 | 0.78 | 0.93 | 0.85 | 0.26 | 0.11 | 0.85 |
| **Pomeranian** | 0.12 | 0.94 | 0.90 | 0.04 | 0.93 | 0.39 | 0.44 | 0.81 | 0.89 |
| **Shih Tzu** | 0.74 | 0.94 | 0.90 | 0.04 | 0.12 | 0.79 | 0.43 | 0.99 | 0.85 |
| **SBT** | 0.73 | 0.91 | 0.44 | 0.004 | 0.051 | 0.74 | 0.75 | 0.99 | 0.28 |
