# Supplementary material for: A cross-sectional study into the prevalence and conformational risk factors of BOAS across fourteen brachycephalic dog breeds
Source: PLoS One. 2026 Feb 18;21(2):e0340604. doi: 10.1371/journal.pone.0340604 (PMC12915975; doi:10.1371/journal.pone.0340604)
Supplement: S5 Table — (DOCX) [file pone.0340604.s006.docx]

| **Breed** | **Odds Ratio** | **95% Confidence Interval** | **P-value** |
| --- | --- | --- | --- |
| Affenpinscher | 0.71 | 1.83e-8 – 1.04e+7 | 0.97 |
| Boston Terrier | 7.3e-15 | 1.11e-22 – 2.46e-8 | <0.0001*** |
| Boxer | 1.8e-11 | 2.02e-21– 4.72e-3 | 0.02* |
| Cavalier King Charles Spaniel | 0.001 | 3.58e-8 – 19.2 | 0.19 |
| Chihuahua | 1.3e-9 | 3.06e-18 – 6.70e-3 | 0.02* |
| Dogue de Bordeaux | 19.3 | 1.54e-4 – 5.79e+6 | 0.62 |
| Griffon Bruxellois | 0.58 | 1.37e-7 – 3.60e+7 | 0.94 |
| Japanese Chin | 0.00 | 1.01e-19 – 3.26e+4 | 0.23 |
| King Charles Spaniel | 66.7 | 1.27e- 8 – 7.53e+11 | 0.71 |
| Maltese | 0.00 | 0 – Inf | 1.00 |
| Pekingese | 5.5e5 | 1.16e-15 – 9.29e+30 | 0.56 |
| Pomeranian | 2.9e-10 | 1.50e-21 – 0.11 | 0.06 |
| Shih Tzu | 9.7e-6 | 3.96e-12 – 3.11 | 0.09 |
| Staffordshire Bull Terrier | 2.2e-4 | 6.80e- 9 – 4.13 | 0.10 |
