# Supplementary figures and images for: A cross-sectional study into the prevalence and conformational risk factors of BOAS across fourteen brachycephalic dog breeds
Source: PLoS One. 2026 Feb 18;21(2):e0340604. doi: 10.1371/journal.pone.0340604 (PMC12915975; doi:10.1371/journal.pone.0340604)

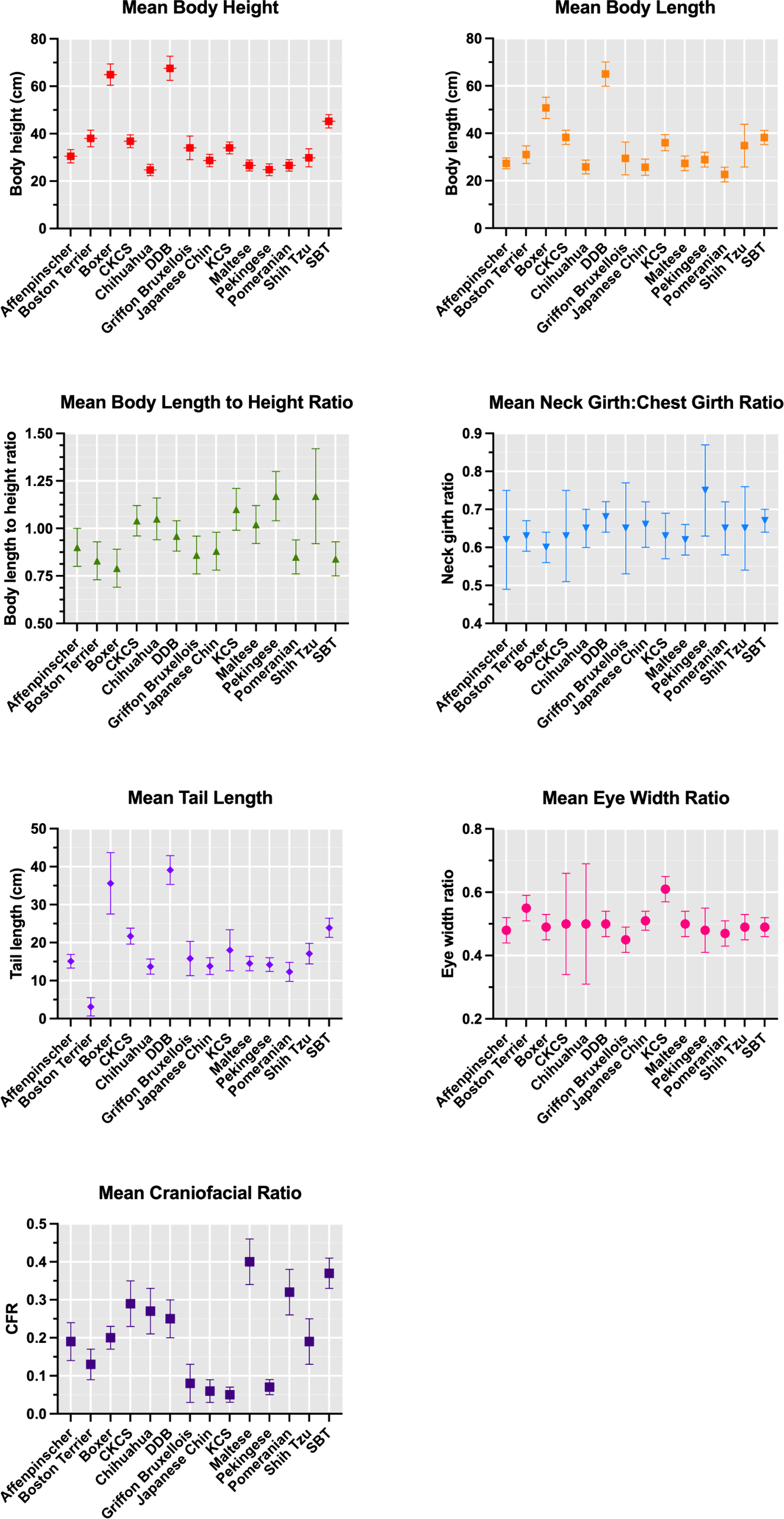

Supplement: S1 Fig — Physical measurements: body height (BH), body length (BL), tail length (TL). Ratios calculated: neck to chest girth ratio (NGR), eye width ratio (EWR) and craniofacial ratio (CFR). (TIF) [file pone.0340604.s007.tif]

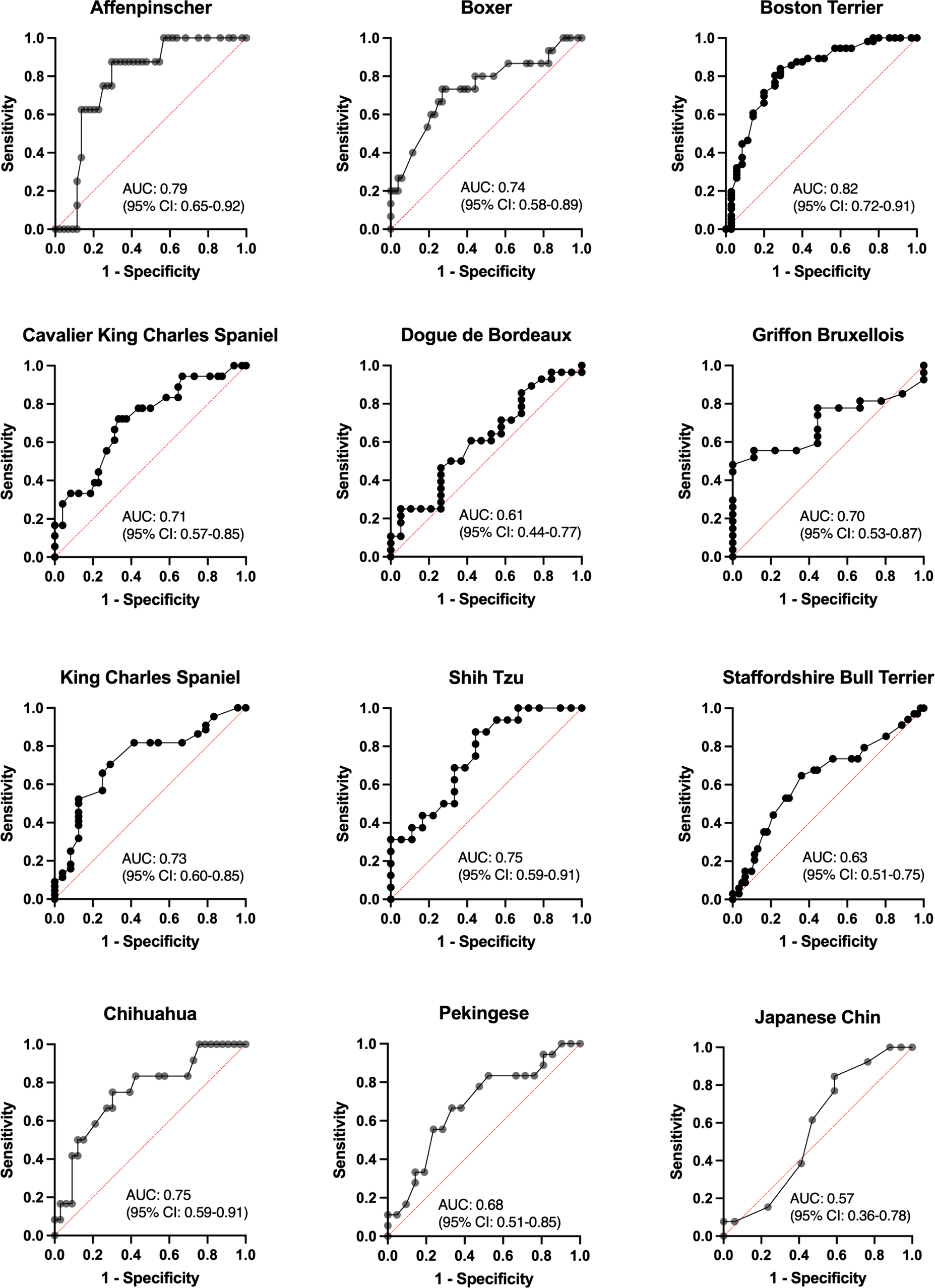

Supplement: S2 Fig — Receiver operator curves (ROC) labelled with the area under ROC curve (AUC) and 95% confidence interval (95% CI). The Chihuahua model excludes the variable nostril stenosis and the Japanese Chin model excludes the variables body condition score and nostril stenosis. (TIF) [file pone.0340604.s008.tif]

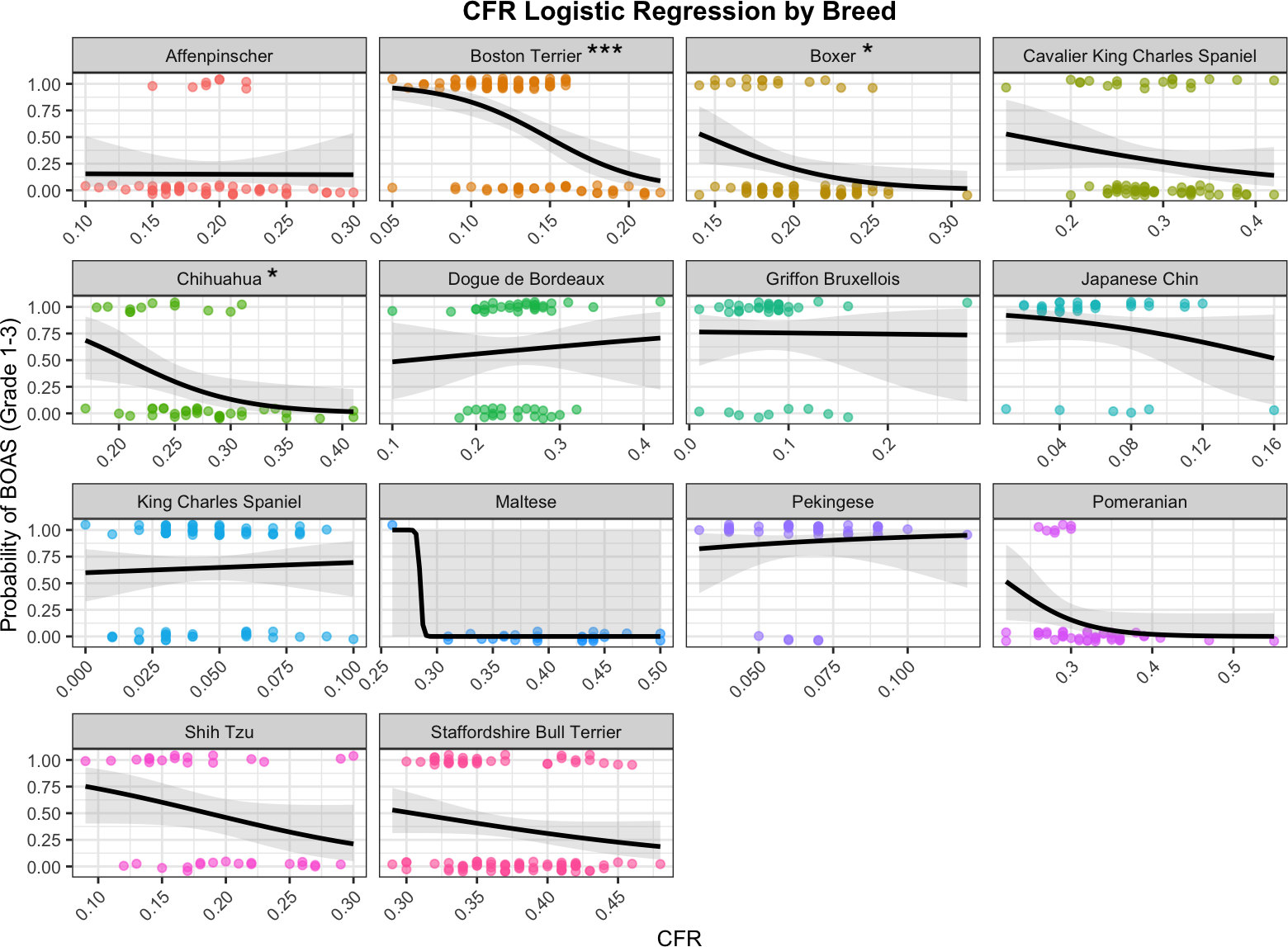

Supplement: S3 Fig — Breeds found to have a significant association between BOAS status and craniofacial ratio include the Boston Terrier (p < 0.0001), Boxer (p = 0.02) and Chihuahua (p = 0.02). (TIF) [file pone.0340604.s009.tif]
